# Clinical Outcome and Histopathologic Assessment of Canine Perivulvar Mast Cell Tumours

**DOI:** 10.1111/vco.70104

**Published:** 2026-08-12

**Authors:** Madison R. Ahrens, Sarah M. Corner, Alison Masyr

**Affiliations:** ^1^ Department of Small Animal Clinical Sciences, College of Veterinary Medicine Michigan State University East Lansing Michigan USA; ^2^ Veterinary Diagnostic Laboratory, College of Veterinary Medicine Michigan State University Lansing Michigan USA

**Keywords:** dogs, mast cell tumour, perivulvar

## Abstract

Anatomic location is a known prognostic factor for canine mast cell tumours (MCTs). Previous publications have reported conflicting results about the clinical significance of MCT location in the inguinal and perineal regions and male genitalia. Hence, the goal of this study is to evaluate the grade, breed distribution and clinical outcome of canine perivulvar MCT. Surgical biopsy reports and associated medical records submitted between 2013 and 2024 to Michigan State University Veterinary Diagnostic Laboratory were retrospectively evaluated. Information regarding age, reproductive status, breed, tumour location, microscopic description, histopathologic grade, and survival was collected. Kaplan–Meier analysis was used to estimate median survival time (MST) with dogs lost to follow‐up (LTF) or alive censored from analysis. Biopsy reports from 64 dogs with 68 perivulvar MCT were evaluated. The most common phylogenetic classification was Mastiff‐Terrier (47%). The majority (97%) of evaluated MCT were cutaneous, with 85% categorised as Kiupel low grade, while the remaining 15% were high grade. Three of 19 tumours (16%) that had *c‐KIT* PCR performed had mutations in either Exon 8 or Exon 11. KIT expression pattern was classified as Pattern 2 or 3 in 71% of evaluated tumours. Median Ki‐67 proliferation index and Ki‐67 × AgNOR scores were 7 and 13, respectively. MST for the entire population was 868 days. Dogs LTF had a median follow‐up time of 442 days. Dogs with perivulvar MCT may achieve prolonged survival time with surgical excision, and prospective studies are warranted to determine the effect of adjuvant therapy on outcome.

## 1 Introduction

Mast cell tumours (MCTs) are the most common skin tumours in dogs, accounting for 11%–27% of malignant skin tumours in dogs [1, 2]. Mast cells originate from pluripotent haematopoietic stem cells in the bone marrow [3, 4]. These cells display a variety of biological, morphological, biochemical and functional characteristics [4]. The likelihood of developing MCT increases with age, with a mean age of tumour development at 8.5 years, though there have been reports of dogs as young as 14 weeks diagnosed with MCT [5]. MCT have been reported in a variety of breeds with a higher occurrence in boxers, Boston terriers and other breeds of bulldog descent [6]. Recent studies have shown that there is a genetic predisposition to developing high‐grade MCT in the French bulldog, pitbull terrier, Rottweiler, Shih Tzu and Chinese shar pei. In contrast, breeds such as the golden retriever, pug and boxer have been shown to develop low‐grade MCT more commonly [6, 7, 8, 9].

There are several known prognostic factors in dogs with MCT. These include: tumour grade, anatomic location, cutaneous versus subcutaneous location, KIT protein expression pattern evaluated by immunohistochemistry (IHC), cellular proliferation indices such as Ki‐67 and argyrophilic nucleolar organiser regions (AgNORs), *c‐KIT* mutation status, duration of tumour growth, and clinical staging [10, 11, 12].

In dogs, 50% of cutaneous MCT are on the trunk and perineal region while 40% are found on the extremities and the remaining 10% are found on the head and neck [6, 13, 14]. Although perineal MCT are common, there are few studies characterising the biological behaviour and clinical outcome in this location. The consensus of these studies is that cutaneous MCT in the perineal and inguinal region, including scrotum, prepuce and vulva, are more likely to recur or metastasise as compared to other cutaneous MCT along the trunk and extremities [15]. Due to anatomic location, achieving complete surgical excision of these tumours can be difficult [15]. MCT of the preputial, inguinal and subungual regions along with oral/perioral regions and other mucocutaneous sites have been known to display a more aggressive behaviour [15, 16, 17]. Recent studies have reported that the median disease‐free interval for dogs with MCT in the inguinal region was 9.6 months, in contrast to 33.9 months for dogs with MCT in other cutaneous locations [15]. Dogs with MCT in the preputial region had an even lower disease‐free interval of 4.2 months [15, 18]. Conversely, an additional recent study found that scrotal and preputial MCT had similar biological behaviour to MCT of other locations [19]. Although the vulva is within the perineal region, MCT of this location have not been solely evaluated. A study evaluating dogs with high‐risk MCT undergoing chemotherapy found that dogs with mucosal MCT experienced significantly shorter median survival times (MSTs) compared with dogs with MCT of haired skin [20, 21]. Given their location in the perineal region and mucocutaneous anatomy, it would stand to reason that perivulvar MCT would also have more aggressive biological behaviour than MCT at other cutaneous sites. However, anecdotally, the author's clinical experience (A.M.) would suggest that perivulvar tumours tend to have more benign biological behaviour. Hence, this study will evaluate the grade, breed distribution and clinical outcome of canine perivulvar MCT exclusively.

## 2 Methods

This retrospective study was conducted by evaluating surgical biopsy reports from biopsy samples submitted to the Michigan State University Veterinary Diagnostic Laboratory (MSU VDL) between January 2013 and August 2024. Search words for inclusion in this study included: ‘vulvar mast cell tumour’ and ‘perivulvar mast cell tumour’. For the purpose of this study, perivulvar location includes any tumour in the perivulvar haired skin, mucocutaneous junction or vulvar mucosa. Information regarding age, reproductive status, breed, tumour location, microscopic description and Kiupel histopathologic grade were acquired from the sample submission forms and surgical biopsy reports [22]. Subcutaneous MCT were classified as equivalent to ‘low grade’ if there was no evidence of infiltrative growth pattern, multinucleation, mitotic count greater than 4 per 2.37 mm^2^, or any other high‐grade features based on the cutaneous two‐tier grading system [12]. Breeds were assigned to one of five major dog breed clusters based on microsatellite clustering analysis previously performed using 130 dog breeds [23, 24]. The five groups are Ancient‐Asian, Herding‐Sighthound, Modern, Mastiff‐Terrier and Mountain. Breeds that did not fall under any of these five groups were classified as ‘no assignable phylogenetic cluster’.

Clinical data and follow‐up information for animals from each surgical biopsy report were obtained via medical records from referring veterinarians. Follow‐up data included: duration of tumour growth, longest tumour dimension, history of other MCT, presenting complaints before perivulvar MCT diagnosis, clinical signs related to perivulvar MCT (including polyuria, polydipsia, lethargy, recurrent urinary tract infection, urinary incontinence and scooting) and date of death, if applicable.

Tumour grade and histopathologic description were obtained from surgical pathology reports written by multiple ACVP board‐certified anatomic pathologists at MSU VDL, and reports were reviewed by one ACVP board‐certified anatomic pathologist (S.M.C.). All neoplasms were assessed by microscope. Mitotic count is typically assessed by finding the most mitotically active region (hot spot) and counting all mitoses in an area equivalent to 2.37 mm^2^: 10 contiguous 400× fields (FN 22 mm ocular). In cases that had additional testing previously performed (including PCR for *c‐KIT* mutation in Exons 8 and 11, KIT pattern expression, Ki‐67 proliferation index, AgNORs/cell, and combined Ki‐67 × AgNOR score), these data were also included. Methods for these additional PCR and immunohistochemical tests, including Ki‐67 and AgNORs proliferation scoring, were performed as previously published [25, 26, 27]. This information was also reported by multiple ACVP board‐certified anatomic pathologists at MSU VDL, and reports were reviewed by one ACVP board‐certified anatomic pathologist (S.M.C.). Additional data obtained from surgical pathology reports included tumour location (haired or non‐haired skin, cutaneous or subcutaneous), presence of ulceration and mitotic count. For those cases that included a lymph node with MCT submission, the grade of nodal metastasis using Weishaar's classification system was also recorded [28]. For assessing completeness of excision, standard biopsies at MSU VDL are trimmed using a halves and quarters method, thereby assessing lateral margins in four radial planes of section, and assessing the deep margin in each of those planes, then making an overall assessment of completeness of excision based on these sections. Margins were characterised as ‘complete’ if deemed so by the interpreting pathologist or considered ‘incomplete’ if the pathologist described neoplastic cells < 1 mm from the evaluated margins.

### 2.1 Statistical Analysis

Survival time was defined as the date the pathology report was submitted to the date the dog was deceased. If only month and year were available for time of death, the first day of that month was assumed. Kaplan–Meier survival analysis was performed to determine MST, median follow‐up time, and confidence intervals (95% CI) using GraphPad Prism 10.2.3. Dogs that were lost to follow‐up (LTF) or alive at the time of analysis were censored.

## 3 Results

### 3.1 Study Population

A total of 64 dogs with 68 perivulvar MCT were identified. The median age at diagnosis was 8 years old. All dogs were female and 93.8% (*n* = 60) were spayed while the remaining 6.2% (*n* = 4) were intact. The most common phylogenetic breed classification was Mastiff‐Terrier, with 46.9% (*n* = 30) falling into this category. The most common Mastiff‐Terrier breeds were American Pit Bull Terrier (*n* = 8, 12.5%), boxer (*n* = 7, 10.9%), Labrador retriever (*n* = 4, 6.3%), Boston terrier (*n* = 3, 4.7%) and French bulldog (*n* = 3, 4.7%). An additional 15.6% (*n* = 10) of dogs were in the modern phylogenetic classification. The most common breeds identified with MCT within this category were pug (*n* = 3, 4.7%) and cocker spaniel (*n* = 3, 4.7%). The remaining 37.5% (*n* = 24) of dogs were grouped as having no assignable phylogenetic cluster, including mixed breed dog (*n* = 18, 28.1%), Weimaraner (*n* = 4, 6.3%), cane corso (*n* = 1, 1.6%) and Rhodesian ridgeback (*n* = 1, 1.6%).

### 3.2 Tumour Grade Classification and Histopathologic Characterisation

Of the 68 tumours in this study, 85.3% (*n* = 58) were classified as low grade while the remaining 14.7% (*n* = 10) of the tumours were high grade. Ulceration was noted in 16.1% (*n* = 11) of the surgical pathology reports, 4 of which were from high‐grade MCT. The overall median mitotic count for all tumours was 0–1 per 2.37 mm^2^. Among the high‐grade tumours, the median mitotic count was 12 per 2.37 mm^2^ (range 2–34). The median mitotic count of low‐grade tumours was 0 per 2.37 mm^2^ (range 0–5).

Based on the histopathologic description from the surgical pathology reports, 97.1% (*n* = 66) of the tumours were located in the dermis, while the remaining 2.9% (*n* = 2) tumours were entirely within the subcutis. MCTs were in haired skin in 85.5% (*n* = 47) of cases, non‐haired skin (mucosal surface) in 5.4% (*n* = 3), and at the mucocutaneous junction in 9.1% (*n* = 5).

Tumour margins were deemed ‘complete’ by the overseeing pathologist in 82.4% (*n* = 56) of cases. Margins were described as ‘incomplete’ or unable to be confirmed in the remaining 17.7% (*n* = 12) of cases.

### 3.3 KIT Expression Pattern and Proliferation Indices

There were 19 tumours with further testing results available (Table 1), 17 of which were classified as low grade. All 19 tumours underwent *c‐KIT* PCR testing. Two tumours had *c‐KIT* mutations in Exon 8 (one high grade and one low grade), and one additional tumour had a *c‐KIT* mutation in Exon 11 (low grade) identified by PCR. KIT expression pattern assessed by IHC was available for 17 tumours, with 70% (*n* = 12) classified as KIT pattern 2 or 3. Excluding the one high‐grade tumour with additional proliferation indices, the median Ki‐67 index score among the evaluated low‐grade tumours was 6.5 (*n* = 14), along with a median AgNOR/cell value of 1.84 and a median combined Ki‐67 × AgNOR score of 12.5. Two high‐grade MCT had further testing. One only had *c‐KIT* PCR performed. The other high‐grade tumour had a KIT pattern of 3 along with a Ki‐67 × AgNOR score of 81.5.

**TABLE 1:** Summary of additional testing results from 19 dogs with perivulvar MCT.

Variable	*N* (%)
*c‐KIT* mutation	*N* = 19
Exon 8	2 (11)
Exon 11	1 (5)
Mutation absent	16 (84)
KIT expression pattern	*N* = 17
Type 1	5 (29)
Type 2	10 (59)
Type 3	2 (12)
Ki‐67 and AgNOR	*N* = 15
Ki‐67	Median: 7
AgNOR/cell	Median: 1.87/cell
Ki‐67 × AgNOR	Median: 13

### 3.4 Lymph Node Analysis

Regional lymph nodes were available for histologic evaluation in 10 dogs. Analysed lymph nodes included: inguinal (*n* = 8) and popliteal (*n* = 2). Of the 10 submitted lymph nodes, 8 were from dogs with low‐grade tumours while the remaining 2 were from dogs with high‐grade tumours. Five (50%) of the lymph nodes were classified as HN0, one (10%) as HN1 and one (10%) as HN2. Three lymph nodes were classified as HN3, including two dogs with high‐grade MCT and one dog with a low‐grade MCT.

### 3.5 Medical History Review

We were able to obtain medical histories for 42 of 64 (66%) dogs. Tumour growth duration information was available for 22 of 42 dogs (52.4%), with 40.9% (*n* = 9) of tumours present for > 6 months and 51.9% (*n* = 13) of tumours present for < 6 months. Tumour dimensions were available for 36 of 42 (85.7%) dogs. The median longest tumour diameter was 1 cm (range 0.3–4.5 cm). Various perivulvar‐associated clinical signs were noted, most commonly urinary issues, in 19% (*n* = 8/42) of the dogs. Reported signs included polyuria, polydipsia, haematuria, recurrent urinary tract infections and urinary incontinence. Eight of the 42 dogs (19%) with full medical histories received anti‐neoplastic therapy including toceranib phosphate (37.5%, *n* = 3), radiation therapy (25%, *n* = 2), vinblastine (25%, *n* = 2) and doxorubicin (12.5%, *n* = 1). One dog had documented perivulvar MCT recurrence at 48 days following the initial surgery. This dog's MCT was characterised as high grade, with a mitotic count of 20 per 2.37 mm^2^ and incomplete surgical margins.

Additional MCT were documented in 88.1% (*n* = 37/42) of dogs, and of those, 91.9% (*n* = 34) were classified as low‐grade tumours while the remaining 8.1% (*n* = 3) were classified as high grade. Among dogs with multiple MCT, 16.2% (*n* = 6) were in the inguinal/perianal region. Based on overseeing clinician assessment, none of the additional MCT documented were suspected to be recurrence of previous MCT based on tumour location.

### 3.6 Survival Analysis

There were 43 dogs available for survival analysis. The MST for dogs with perivulvar MCT was 868 days (95% CI: 849–884) (Figure 1). Twenty dogs were immediately LTF after surgery; these dogs were assigned a survival of 1 day and censored from the analysis. An additional 19 were censored: 13 LTF with > 1 day known survival and 6 alive at the time of analysis. For the 13 dogs LTF, the median follow‐up time was 442 days (95% CI: 415–465). For the 6 dogs that are still alive, the median follow‐up time was 359 days (95% CI: 321–389).

**FIGURE 1:**
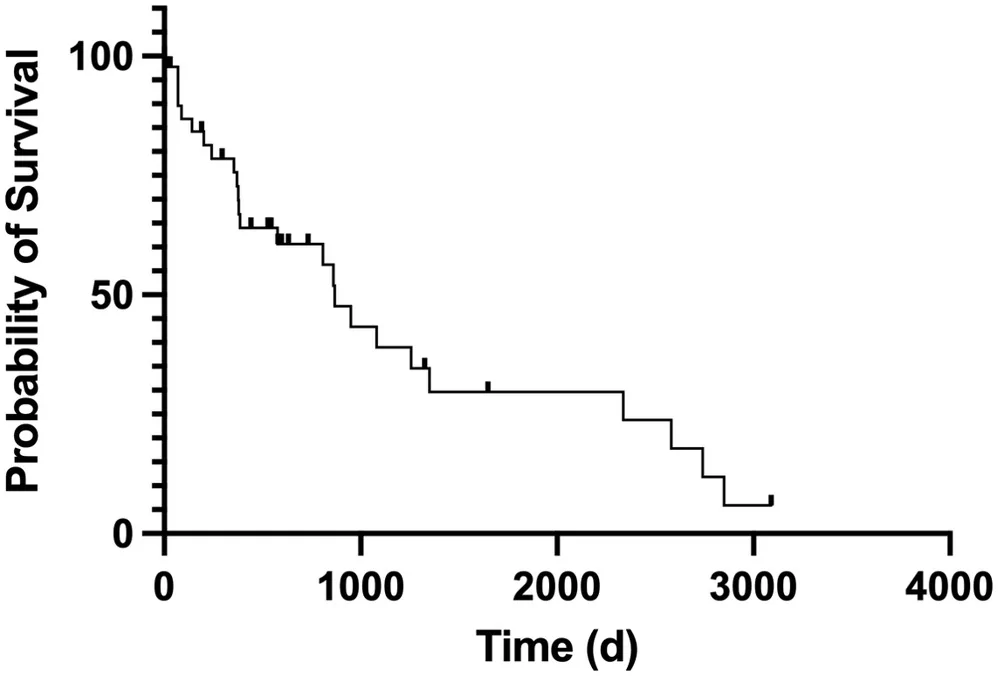
Kaplan–Meier survival curve. Canines with perivulvar MCT had an MST of 868 days. Individuals with < 1 day of known follow‐up were excluded, and those LTF or alive at the time of analysis were censored.

## 4 Discussion

This is the first study to exclusively evaluate canine perivulvar MCT. In line with other studies, the breed distribution of dogs was similar to that of dogs with MCT in other cutaneous locations. Specifically, dogs of bulldog descent, such as those clustered into the Mastiff‐Terrier group, were the most common in this population. Sfiligoi et al. reported the median age of dogs with inguinal or perineal MCT was 8 years [15]. Similarly, the median age of dogs with perivulvar MCT in the present study was approximately 8 years.

While inguinal MCT has been reported to be predominantly high grade [29], the majority of perivulvar MCT in this study were low grade and had low proliferation indices confirmed by Ki‐67 and/or AgNOR scores in 17 tumours. Ki‐67 is a protein expressed throughout the cell cycle, while AgNOR indicates the number of organisational units within the nuclei and correlates with the speed of cellular proliferation [26, 30]. Studies have shown that a Ki‐67 × AgNOR product score above the cut‐off value is associated with increased risk of mortality and metastasis [26, 30]. The 14 low‐grade MCT with additional testing performed had a median Ki‐67 index of 6.5 and a median Ki‐67 × AgNOR combined score of 12.5, both of which are below the respective cut‐off values associated with worsened prognosis and decreased survival times (Ki‐67 < 23, Ki‐67 × AgNOR scores < 54) [26]. The low Ki‐67 × AgNOR scores in this subset further support our conclusion that perivulvar MCT are commonly classified as low grade.

Mutations in *c‐KIT* are a prognostic indicator for dogs with cutaneous MCT, with Exons 8 and 11 most commonly affected [31, 32, 33]. Mutations in Exon 11 occur in 20%–45% of MCT and have been associated with aberrant KIT protein localisation, high tumour grade, and increased proliferative activity as demonstrated by Ki‐67 index along with Ki‐67 × AgNOR scores [25, 31, 32, 33, 34, 35, 36]. Dogs with *c‐KIT* mutations in Exon 11 are at greater risk of recurrent disease and MCT‐related mortality [25, 32, 36, 37, 38, 39]. Only 5% (1/19) of evaluated tumours in this study demonstrated a mutation in *c‐KIT* exon 11. Notably, this was a low‐grade tumour and neither of the two high‐grade tumours that underwent *c‐KIT* PCR had a mutation at this exon. Unfortunately, this dog was LTF immediately following surgery. Approximately 33% of MCT contain mutations in Exon 8 of *c‐KIT*; however, these dogs often have a better prognosis and tumours are often of lower histological grade with lower proliferative activity and less aberrant KIT localisation [25, 36, 38]. Only 11% (2/19) of evaluated tumours in this study demonstrated a mutation in *c‐KIT* Exon 8, representing one high‐ and low‐grade tumour each.

Aberrant KIT expression identified by IHC can be associated with poorer prognosis in dogs with MCT. KIT localisation within the cell demonstrates three distinct labelling patterns: (1) membrane‐associated, (2) focal to stippled perinuclear cytoplasmic localisation accompanied by decreased membrane‐associated localisation and (3) diffuse cytoplasmic labelling [27, 40]. KIT patterns 2 and 3 are associated with poorer prognosis [27]. Interestingly, in the present study, 70% (12/19) of tested MCT exhibited KIT pattern 2 or 3. This finding is surprising as most of these were low‐grade MCT in dogs that achieved prolonged survival. While the number of tumours evaluated is small, this finding suggests that KIT pattern may not be as closely linked with prognosis in perivulvar MCT as in MCT of other locations.

The HN0–HN3 system is a classification scheme for MCT‐associated lymph nodes that is based on the intranodal quantity and distribution of mast cells and degree of disruption of nodal architecture. This system has been shown to correlate with clinical outcomes and prognosis in dogs with MCT [28]. Among the 10 lymph nodes submitted for biopsy along with the perivulvar MCT, 70% (*n* = 7) were classified as HN0–HN2. Although a study performed by Weishaar et al. found that dogs with lymph nodes classified as HN2/3 had a worse clinical outcome compared to lymph nodes classified as HN0/1, a more recent study found that HN2 lymph nodes can be associated with prolonged survival and these dogs may not warrant systemic treatment [28, 41]. Consistent with tumour grade, both cases with high‐grade tumours that underwent lymph node evaluation were classified as HN3.

Tumour ulceration has been known to be an indicator of poor prognosis in cutaneous MCTs [10, 21]. Tumour ulceration was an uncommon finding in this population, with 83.8% of tumours (57/68) having no histologic evidence of ulceration. MCT growth duration was only available for 24 tumours, with 41.7% (*n* = 10) growing for more than 6 months, and 58.3% (*n* = 14) growing for less than 6 months. While short duration of MCT growth is associated with more aggressive tumours, it is possible that perivulvar MCT are identified more quickly by clients given that 20% of dogs in this study displayed urinary‐related clinical signs that may prompt rapid evaluation by a veterinarian. It is already well‐known that dogs with MCT often develop additional MCT [10, 42, 43]. Dogs in the present study followed this trend, with nearly 60% having a history of cutaneous MCT at other locations. Similar to the perivulvar MCT, additional MCT in these dogs were overwhelmingly classified as low grade. Despite the challenging anatomical location, over 80% of tumours were described as completely excised by the reviewing pathologist, and only one dog—with an incompletely excised, high‐grade MCT—was documented as having recurrent disease. This low recurrence rate is consistent with that of other publications [44, 45, 46, 47].

Finally, the MST for dogs in this study was 868 days. Despite the setback of not knowing if or when some of the dogs died, the dogs LTF had a median follow‐up time of 442 days. For the 9 dogs still alive at the time of publication, the median follow‐up time was 191 days. All of these dogs underwent excisional biopsy at minimum. Full medical histories revealed that 20% of dogs received some sort of anti‐neoplastic therapy. Because of the small sample size, we did not attempt to determine if adjuvant treatment impacted survival.

This study has several limitations, mostly as a consequence of its retrospective design. Differential losses to follow‐up and medical histories from referring veterinarians with a variety of styles of documentation and thoroughness certainly impacted completeness of our dataset. We attempted to offset the impact of missing data as best as possible, for example, by assuming a first of the month death date for dogs with only month and year recorded. This may have slightly underestimated MST, though the effect is believed to be negligible given the prolonged MST that was calculated. Due to the limited and variable medical records, we were unable to determine if any dogs underwent additional staging tests following MCT diagnosis. While this study utilised biopsy reports from ACVP board‐certified anatomic veterinary pathologists, there is some possibility of interpathologist disagreement on tumour grading. Similarly, only one dog had documented perivulvar MCT recurrence, though this may not accurately reflect the true recurrence rate. This is additionally why we focused on survival time rather than including disease‐free interval. The aim of this study was to characterise clinical and histopathologic data related to perivulvar MCT. While we are pleased with a sample size of 68 tumours, we did not perform statistical analysis on the effect of said clinical or histopathologic data as the resultant sample sizes would hinder developing meaningful conclusions.

This study is the first to focus specifically on canine perivulvar MCT and our findings suggest that these tumours are generally less aggressive than other MCT in the inguinal or perineal region. Affected dogs had similar signalment to dogs with cutaneous MCT overall, with Mastiff‐Terrier breeds predominating. Most perivulvar MCT were low grade, further supported by low Ki‐67 indices, low Ki‐67 × AgNOR scores, low frequency of *c‐KIT* mutations and tumour ulceration. The most common KIT staining patterns were those associated with decreased disease‐free interval, though our population experienced prolonged survival (> 2 years) following surgery, suggesting that KIT localisation may be a less reliable prognostic indicator for perivulvar MCT. Additional studies would be needed to further define prognostic factors specific to this tumour location.

## Funding

The research reported in this publication was supported by the Office of the Director of the National Institutes of Health under Award Number T35OD016477. The content is solely the responsibility of the authors and does not necessarily represent the official views of the National Institutes of Health.

## Disclosure

The authors have nothing to report.

## Conflicts of Interest

The authors declare no conflicts of interest.

## Data Availability

The data that support the findings of this study are available from the corresponding author upon reasonable request.
